# Lamellar Macular Holes: The Role of Microperimetry in Functional Assessment

**DOI:** 10.1155/2019/9035837

**Published:** 2019-04-14

**Authors:** Simone Donati, Paola Della Valle, Elias Premi, Marco Mazzola, Laura Lo Presti, Claudio Azzolini

**Affiliations:** Department of Medicine and Surgery, University of Insubria-ASST Sette Laghi, Varese, Italy

## Abstract

**Introduction:**

The aim of our observational cross-sectional study was to evaluate the association between visual function and anatomical characteristics of LMH, considering in particular different subtypes of LMH and their features.

**Materials and Methods:**

This observational clinical study has been conducted in the Ophthalmology Clinic, ASST-Sette Laghi, University of Insubria of Varese-Como, Italy. Included patients underwent a complete ophthalmological examination, as well as MP1 microperimetry evaluation and optical coherence tomography (OCT). Two experienced masked observers evaluated OCT imaging in order to assess the integrity of the photoreceptor layer (interdigitation zone and ellipsoid zone: IZ/EZ) and the external limiting membrane (ELM).

**Results:**

Twenty-five patients affected by an LMH were evaluated. Eighteen eyes of 18 patients met the study criteria and were included. Based on morphological and functional data, LMHs were divided into two subgroups: tractional (tLMH) and degenerative (dLMH). We identified 11 tLMHs and seven dLMHs. Functional parameters showed a significative difference in visual acuity and retinal sensitivity between the two groups, respectively: (sample median and the interquartile range) 0.0 (0.0; 0.09) LogMAR vs 0.15 (0.09; 0.52) LogMAR and 16.2 (14.2; 17.7) dB vs 10.0 (7.5; 11.8) dB (*p* < 0.05). Fixation was predominantly central in 90.9% of tLMH vs 71.4% of dLMH and stable in 81.8% tLMH vs 42.9% dLMH, but the differences were not statistically significant. Tractional and degenerative LMHs showed no significant differences in central foveal thickness. Conversely, LMH depth and horizontal diameters appeared different between the two groups. Tractional LMH showed a greater depth 257 (205; 278) *μ*m vs 190 (169; 249) *μ*m, whereas degenerative LMH showed a greater horizontal diameter 653 (455; 750) *μ*m vs 429 (314; 620) *μ*m (*p* < 0.05). IZ/EZ line was unaffected in 81.8% of tLMHs eyes versus 14.3% of dLMHs eyes (*p* < 0.05). Visual acuity and retinal sensitivity were higher in eyes with integrity of both IZ/EZ and ELM compared to those with a disruption of one or both layers (*p* < 0.05).

**Conclusion:**

Two different subtypes of LMH showed peculiar functional aspects due to their morphological features. Tractional LMHs revealed higher visual acuity and retinal sensibility due to the relative preservation of the outer retinal layers compared to degenerative LMHs. Moreover, we underlined the importance of microperimetry to better identify functional defects in macular degenerative pathologies.

## 1. Introduction

Lamellar macular hole (LMH) is a retinal pathology characterized by a morphologic alteration of the structure of the fovea, which could lead to metamorphopsia and a reduction in best-corrected visual acuity (BCVA) [1, 2].

Spectral-domain OCT (SD-OCT) provides an excellent visualization of the retinal structure and retinal layers [3]. Its role in characterizing LMHs has been fundamental for investigating subgroup characteristics and visualizing pathological lesions such as epiretinal membranes and proliferations [1, 4].

Recently, the International Vitreomacular Study Group suggested optical coherence tomography (OCT) features to identify LMHs: defect in inner fovea; irregular foveal contour; intraretinal schisis; and preservation of the ellipsoid zone (EZ). The pathogenesis of LMHs still has to be fully understood, as well as its indication for surgical treatment [5].

Microperimetry is used to test sensitivity to light stimulus, with a precise point-to-point analysis. An eye-tracking system is used to correct noncentral fixation, and an infrared fundus image is constantly and simultaneously provided along the light sensitivity test. Microperimetry is of particular importance to assess the functional status of the macula in those pathologies characterized by subclinical symptoms, such as LMHs [6, 7].

The aim of our study was to evaluate the association between visual function and anatomical characteristics of LMH, considering in particular different subtypes of LMH and their features.

## 2. Materials and Methods

In this observational cross-sectional study, we included consecutive patients with a diagnosis of an LMH examined at the Vitreoretinal Outpatient Service, Ophthalmology Clinic, University of Insubria of Varese, Italy, between September 2017 and July 2018.

**Figure 1:**
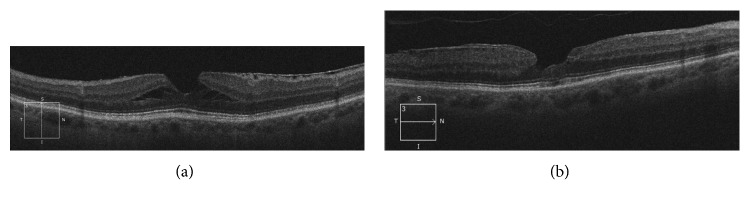
SD-OCT evaluation of tractional (a) and degenerative (b) lamellar macular hole.

An LMH was diagnosed based on SD-OCT characteristics as proposed by Witkin and as classified by Duker [2, 3]. Inclusion criteria were as follows: (1) break in the inner fovea; (2) irregular foveal contour; (3) separation of the inner/outer foveal retinal layers, leading to an intraretinal split; (4) absence of a full-thickness foveal defect (Figure 1).

Exclusion criteria were as follows: (1) the presence of myopia of more than three diopters in the affected eye (axial length more than 25 mm if pseudophakic eyes); (2) retinal pathologies that could influence a correct diagnosis or functional evaluation, such as age-related macular degeneration (AMD), diabetic retinopathy or retinal vascular occlusion; (3) vitreous hemorrhage; (4) cataract graded more than N03 or NC3, according to the Lens Opacity Classification System; (5) ocular surgery other than uncomplicated cataract surgery. Patients presenting low-quality SD-OCT imaging or unable to perform microperimetry evaluation due to weak cooperation were excluded.

All subjects underwent a complete ophthalmologic examination along with intraocular pressure measurement. Instrumental examination included microperimetry, and SD-OCT evaluation. Our study followed the methods published on Reibaldi et al. [8].

All subjects signed an informed consent for clinical examination and data management. Hospital ethical committee considered all clinical procedures as standard evaluation not requiring specifically intended approval.

BCVA was measured by Snellen charts and then converted to LogMAR for statistical analysis.

**Figure 2:**
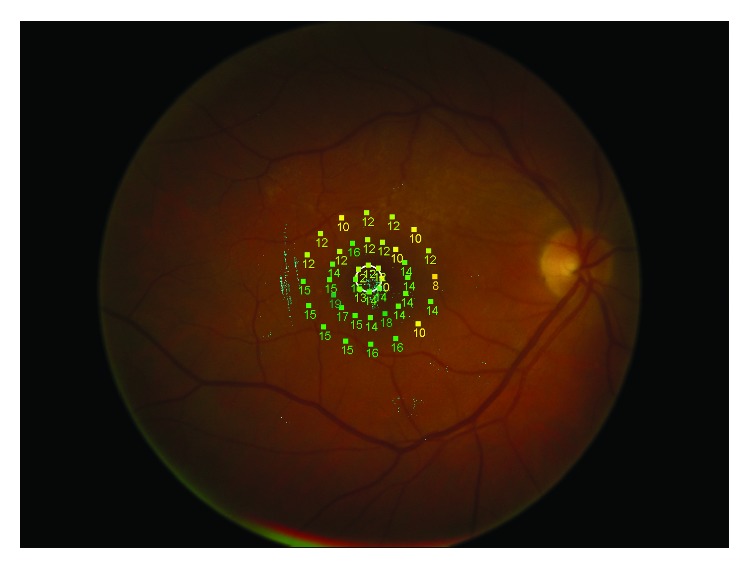
Fundus color picture with retinal sensitivity grid.

An MP-1 microperimeter (Nidek Technologies, Padua, Italy) was used to test retinal sensitivity and fixation. After the pupils were dilated (1% tropicamide), a reference frame was obtained with the integrated infrared camera. We used a 4-2-2 double-staircase test strategy with white background illumination set at 4 apostilbs and a starting stimulus light attenuation set at 10 dB. A grid of 45 stimuli with a Goldmann III stimulus size and a time between the stimuli of 1 s was projected onto the central 8° (Figure 2). A bright red cross of 2° was used for the fixation target. For the assessment of fixation, the fundus movements were tracked during examination.

The mean retinal sensitivity (total sensitivity, mTRS) and the mean sensitivity of the central 13 points within 2° (mean central sensitivity, mCRS) were calculated. The fixation pattern was evaluated as fixation stability and fixation location. Fixation stability was divided into three categories: stable, relatively unstable, or unstable. If 75% of fixation points were located within a two-degree diameter circle, regardless of their position in relation to the foveal center, the fixation was classified as stable. If 75% of fixation points were located within a two-degree circle, but 75% of fixation points were located within a four-degree circle, the fixation was classified as relatively unstable. If 75% of fixation points were located within a four-degree circle, the fixation was classified as unstable. Fixation location was divided into three categories: central, pericentral, and eccentric. If 50% of fixation points were within 0.5 mm of the foveal center, the fixation was classified as central. If 25% to 50% of the fixation points were within 0.5 mm of the foveal center, the fixation was classified as pericentral. If 25% of fixation points were within 0.5 mm of the foveal center, the fixation was classified as eccentric (as in the work of Donati et al. [9]).

To rule out potential learning effects, all patients performed a preliminary test microperimetry examination. All imaging sessions were performed after 5 min of visual adaptation. The same experienced ophthalmologists carried out the examinations (P.D.; L.L.).

SD-OCT images were obtained with a Zeiss Cirrus HD OCT 500 version 7.0.1.290 (Carl Zeiss Meditec, Jena, Germany). All OCT examinations were carried out by a certified operator (S.D., CORC certification 2017). According to the protocol, OCT macular cube 512 × 128 and five-line scans, centered on the fovea, were obtained for each eye. More than 15 scans were averaged for each measurement. Only images with a quality score of more than five were selected as high-quality images.

**Figure 3:**
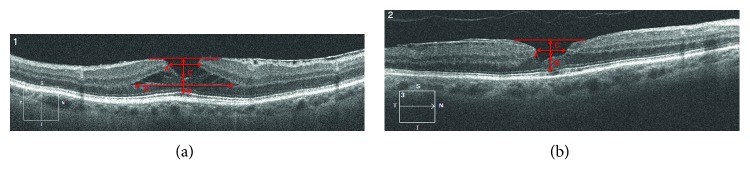
OCT Morphological parameters analyzed in tractional (3.1) and degenerative LMH (3.2): horizontal diameter (A); central foveal thickness (B); depth of LMH (C); base diameter (D).

According to the morphology of the LMH, all included eyes were divided into two subgroups based on the classification published by Govetto et al. [10]: tractional LMH and degenerative LMH (Figures 1 and 3).

The following dimensional parameters of LMHs were measured in *µ*m on grey-scale SD-OCT images by means of a Cirrus software inbuilt manual caliber: horizontal diameter, base diameter, depth of LMH, and central foveal thickness (CFT) (Figure 3). Further analysis was applied considering the integrity of the photoreceptor layer (interdigitation zone and ellipsoid zone: IZ/EZ) and the external limiting membrane (ELM). Retinal layers were evaluated on five-line scans centered on the fovea; they were defined as intact when the line was continuous and disrupted when the line was interrupted by gaps larger than 30 *µ*m. Based on the integrity of two lines, eyes were divided into three groups: Group A = integrity of ELM and IZ/EZ; Group B = integrity of ELM and disruption of IZ/EZ; Group C = disruption of both layers. Two masked expert investigators (E.P.; M.M.) interpreted the SD-OCT images. In case of disagreement, a third investigator (C.A.) was consulted for a final decision.

Continuous variables were summarized using the sample median and the interquartile range due to the low number of observations and the skewed distribution of most parameters. Stable fixation and predominantly central fixation were dichotomized as yes vs. no and summarized using absolute and relative frequencies. To test the null hypothesis of no difference in functional and morphological parameters between the patients' populations with tractional and degenerative LMH, we used the Wilcoxon rank test and Fisher's exact test for continuous and dichotomic variables, respectively. We adopted the same descriptive and inferential approaches to investigate differences in patients' populations defined according to the presence of interruptions in the ELM and IZ-EZ segment. All the analyses were conducted using the SAS software, 9.4 release.

## 3. Results

Twenty-five eyes affected by an LMH were evaluated: of these, seven eyes were excluded (three due to the presence of concomitant macular diseases, two due to excessive refractive error, one due to a significant cataract, and one due to previous vitreoretinal surgery). Therefore, 18 eyes of 18 patients met the study inclusion criteria and were enrolled. Demographic and main clinical characteristics of the enrolled patients are reported in Table 1.

In Table 2, we report morphological and functional data eyes divided into two subgroups: tractional and degenerative LMH. We identified 11 tractional LMHs and seven degenerative LMHs.

Functional parameters showed a significative difference in both visual acuity (*p*=0.03) and retinal sensitivity between the two groups. In particular, we observed a significative difference in both central (*p*=0.0008) and total retinal sensitivity (*p*=0.0001) between tractional and degenerative LMHs (Table 3). Fixation status and stability were different in patients affected by tractional compared to degenerative LMH. Fixation was predominantly central in 90.9% vs 71.4% of eyes and stable in 81.8% vs 42.9% of eyes, respectively, but the differences were not statistically significant.

Considering morphological parameters, tractional and degenerative LMHs showed no significant differences in central foveal thickness: 170 (160; 186) *μ*m vs 157 (112; 175) *μ*m, respectively. Conversely, LMH depth and horizontal diameters appeared different. Tractional LMHs showed a greater depth 257 (205; 278) *μ*m vs 190 (169; 249) *μ*m, whereas degenerative LMHs showed a wider horizontal diameter 653 (455; 750) *μ*m vs 429 (314; 620) *μ*m (*p* < 0.05).

Table 4 shows collected data according to ELM and IZ/EZ integrity. We identified 10 eyes with integrity of ELM and IZ/EZ and 8 eyes with disruption of IZ/EZ associated or not to ELM interruption. Data analysis showed a statistically significant difference in visual acuity and retinal sensitivity between these two groups.

In particular, visual acuity decreases in presence of IZ/EZ disruption: 0.00 (00; 0.09) LogMAR vs 0.15 (0.07; 0.41) LogMAR, respectively. Retinal sensitivity showed the same trend: both mCRS and mTRS decreased from 16.4 (15.7; 17.7) dB to 10.1 (7.8; 11.2) dB and from 15.8 (14.9; 16.7) dB to 12.0 (8.8; 14.8) dB, respectively (*p* < 0.05).

Considering fixation parameters, the eyes showing integrity of both layers present a more frequent stable fixation and a predominantly central fixation status. These data, however, did not reach a statistical significance, probably due to the relatively small sample size.

Considering the morphological parameters of macular hole, the disruption of retinal layers is associated with a reduction in CFT, respectively, 173 (162; 186) *μ*m vs 153 (125.0; 169.5) *μ*m. LMH depth and central foveal thickness did not show significant correlations with IZ/EZ and ELM status.

Considering the integrity of IZ/EZ and ELM in both tractional LMHs and degenerative LMHs, we found that more than 81% of tLMHs present a preservation of external layers compared to 14.2% of dLMHs (*p*=0.0128).

**Figure 4:**
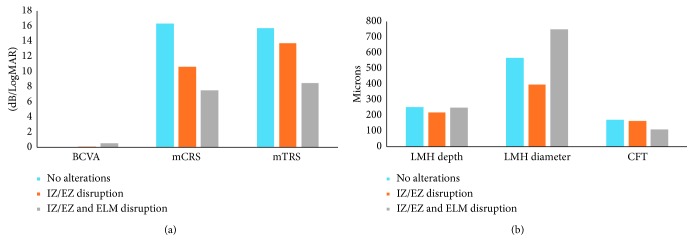
Distribution of eyes according to IZ/EZ and ELM integrity, analyzing functional and morphological parameters. Best-corrected visual acuity (BCVA) in LogMAR; mean central and total retinal sensitivity (mCRS and mTRS) in dB; LMH depth, LMH diameter and central foveal thickness (CFT) in microns.

Figure 4 shows patients' distribution according to IZ/EZ and ELM integrity, analyzing functional and morphological parameters.

## 4. Discussion

Nowadays, LMHs represent a defined macular pathology, classified inside the large chapter of vitreomacular pathologies secondary to an alteration to the vitreoretinal interface [1–3].

The gold standard for the diagnosis and clinical characterization of LMHs is currently OCT imaging, which provides not only qualitative but also quantitative data on this pathology.

Govetto et al. in 2016 defined tractional and degenerative subtypes of LMH by means of OCT examination [10]. The first type is characterized by the schitic separation of the neurosensory retina between the outer plexiform and outer nuclear layers; it presents an intact ellipsoid zone and is associated with tractional epiretinal membranes and/or vitreomacular traction. The second type presents intraretinal cavitations, which could affect all retinal layers; it is associated with nontractional epiretinal proliferation and a retinal “bump”; it often presents with an early ellipsoidal zone defect and its pathogenesis, although chronic and progressive, remains poorly understood.

LMH subgroups were characterized by the same origin, but with different structure and evolution, in particular due to the evidence of an epiretinal tissue proliferation [11]. Published studies identified different characteristics of excised epiretinal membrane (ERM), defined as dense or tractional based on their appearance and behaviour on the retina. They showed different collagen structures as well as different immunoreactivity to glial or smooth muscle actin proteins [12, 13].

In our study, we combined a detailed description of OCT retinal modifications in LMHs with a complete functional evaluation by means of visual acuity and microperimetry examination. Microperimetry is able to quantify foveal and perifoveal retinal sensitivity in an exact fundus-related modality, thus adding detailed information regarding the degree and pattern of macular alteration. The importance of microperimetry was recently underlined by our group into two published clinical studies, in which we investigated the correlation between morphological modifications, retinal sensibility, and fixation status in patients who underwent surgery for epiretinal macular membranes and in patients treated with an intravitreal slow-releasing steroid implant for retinal vein occlusion [9, 14].

The present study underlines the morphological differences between tractional and degenerative LMH. The presence of a tractional ERM in the tLMHs increases LMH depth (median 257 *μ*m vs 190 *μ*m, *p*=0.06) and produces an intraretinal schisis that we measure as LMH base (Figure 3). As previously described, the schisis changes according to OCT scan's orientation, so we did not consider it for statistical purposes.

Degenerative LMHs present larger LMH diameter than tLMHs probably due to progressive retinal degeneration and less tangential traction. Conversely, CFT was similar, despite a different morphology of the foveola (foveal bump in degenerative LMHs and foveolar sparing in tractional LMH).

Considering visual function, we showed a difference between tractional and degenerative LMHs, reflecting different morphological characteristics, as we showed above. Visual acuity, total and central retinal sensitivity appeared significantly higher in tractional LMHs. Considering stability and status of the fixation, tractional LMHs eyes show a prevalent central (90% of eyes) and stable (81.8% of eyes) fixation compared to degenerative LMH eyes. The ability of patients to maintain stability of fixation ensures high quality of visual function while reading or for near activities, as documented also in the case of macular pucker and macular hole, in pre- and postsurgery follow-up [9, 15].

As the second step, we evaluated the integrity of the outer retinal layers, which represents a pathognomonic sign of visual acuity preservation. Several authors have described impaired visual recovery in patients affected by diabetic macular edema or exudative AMD when the IZ/EZ was damaged [16, 17]. Tractional LMHs showed integrity of the IZ/EZ line in 81.8% of the eyes; conversely, in degenerative LMH, IZ/EZ was present only in 14.3% of eyes (*p* < 0.05). Moreover, we documented an alteration of the ELM, which represents the direct connection between photoreceptor and intraretinal architecture. A damage to the ELM, as reported in the literature, is consecutive to that of the IZ/EZ [18], and indicates that morphological changes are not limited to the photoreceptor junction level but extend toward the Muller cell cone. Parravano et al. showed similar results: they compared two type of LMH considering only the aspect of ERM and concluded that the dense not tractional ERM (corresponding to dLMH) correlates with outer retinal layers degeneration. The authors underline, as in our study, the influence of IZ/EZ and ELM on visual acuity and retinal sensitivity preservation [19].

To better understand the role of ELM and IZ/EZ, we divided patients according to outer retinal layers integrity (Table 4). We showed that eyes with no alterations of ELM and IZ/EZ had higher visual acuity, central retinal sensitivity, and stable fixation status (80% of eyes). Eyes presenting a damage to IZ/EZ associated or not to ELM disruption showed a significantly lower visual acuity and retinal sensitivity. As already reported by Reibaldi et al. [8], we showed that retinal morphological characteristics are correlated to central retinal sensitivity more than visual acuity, probably due to the different investigation of visual function. This suggests that microperimetry could be more sensitive in identifying morphological alteration of photoreceptor layer [15].

Tractional and degenerative LMHs present different morphological features due to specific ERM characteristics. Despite inner modification of the fovea due to tractional schisis or horizontal traction with foveal bump, the visual function is influenced by outer retinal layers alterations that involve the photoreceptor complex and cause qualitative and quantitative visual impairment.

Early identification of these alterations may be useful to retina experts for LMHs follow-up or to evaluate the surgical approach.

Limitation of our study was the relatively small patient population, influenced by the low prevalence of this type of pathology and its subclinical symptoms. High-resolution OCT and deep functional analysis (OCT and superimposed microperimetry) may allow us to effectively characterize patients and evaluate their clinical status. A prospective study could be helpful in order to investigate clinical progression of different LMH subtypes and to evaluate the opportunity for surgical intervention [20, 21].

## 5. Conclusions

Our research revealed interesting elements about LMHs: tractional and degenerative LMHs show distinctive functional features that reflect their morphological differences. In particular, tractional LMHs revealed higher visual acuity and retinal sensitivity due to the relative preservation of the outer retinal layers compared to degenerative LMHs.

In order to correctly evaluate foveal degenerative pathologies with slow progression, such as LMH, a multimodal imaging is of fundamental importance. High-resolution OCT associated with microperimetry reveals the morphological and functional modifications of the retina.

## Figures and Tables

**Table 1:** Demographics and main clinical data of enrolled patients.

Patient	Age	Study eye	Funduscopic examination		Visual acuity	
			Study eye	Fellow eye	SE	FE
1	70	RE	Degenerative lamellar macular hole	No abnormalities	0	0
2	68	LE	Degenerative lamellar macular hole	Macular pucker	0.09	0.3
3	75	RE	Tractional lamellar macular hole	Previous surgery for macular hole	0	0.6
4	71	LE	Degenerative lamellar macular hole	Previous surgery for macular pucker	0.5	0.6
5	73	RE	Tractional lamellar macular hole	Macular membrane	0	0
6	82	RE	Degenerative lamellar macular hole	Previous surgery for macular hole	0.15	0.6
7	72	RE	Tractional lamellar macular hole	VMT	0	0
8	68	LE	Tractional lamellar macular hole	No abnormalities	0	0
9	68	LE	Tractional lamellar macular hole	No abnormalities	0	0
10	67	RE	Tractional lamellar macular hole	No abnormalities	0.15	0.09
11	76	LE	Degenerative lamellar macular hole	No abnormalities	0.09	0.04
12	63	RE	Tractional lamellar macular hole	Macula pucker	0.15	0
13	71	RE	Degenerative lamellar macular hole	No abnormalities	0.5	0.15
14	78	LE	Degenerative lamellar macular hole	Macular pucker	0.15	0.6
15	77	RE	Tractional lamellar macular hole	No abnormalities	0.09	0.15
16	74	RE	Tractional lamellar macular hole	Retinal vein occlusion	0	0.04
17	80	LE	Tractional lamellar macular hole	No abnormalities	0.09	0.04
18	76	RE	Tractional lamellar macular hole	No abnormalities	0.04	0.04

Age: years; RE: right eye; LE: left eye; VMT: vitreomacular traction; SE: study eye; FE: fellow eye.

**Table 2:** Morphological and functional data for both studied groups.

Patient	Age	BCVA	mCRS	mTRS	LMH depth	LMH base	LMH diameter	CFT	Integrity ELM	Integrity IZ-EZ	Fixation stability	Fixation status
*Tractional LMH*												
1	75	0.00	17.90	15.85	287	844	537	186	+	+	Stable	Predominant central
2	73	0.00	16.21	16.67	310	1325	650	193	+	+	Relatively instable	Predominant central
3	72	0.00	14.21	13.82	202	1280	601	165	+	+	Instable	Poorly central
4	68	0.00	15.75	16.30	278	560	410	170	+	+	Stable	Predominant central
5	68	0.00	16.62	15.50	205	672	200	232	+	+	Stable	Predominant central
6	67	0.15	15.72	15.65	257	1201	620	176	+	+	Stable	Predominant central
7	63	0.15	11.75	17.25	268	1190	346	185	+	−	Stable	Predominant central
8	77	0.09	16.51	14.85	254	1287	634	162	+	+	Stable	Predominant central
9	74	0.00	18.62	18.06	167	499	243	160	+	+	Stable	Predominant central
10	80	0.09	17.65	17.00	263	1946	429	157	+	+	Stable	Predominant central
11	76	0.04	10.25	15.68	217	1107	314	138	+	−	Stable	Predominant central

*Degenerative LMH*												
1	70	0.00	12.75	13.77	222	nd	397	164	+	−	Instable	Predominant eccentric
2	68	0.09	11.75	12.45	169	nd	653	186	+	+	Stable	Predominant central
3	71	0.52	8.00	14.00	249	nd	750	103	−	−	Instable	Poorly central
4	82	0.30	7.50	8.50	190	nd	678	149	−	−	Relatively instable	Predominant central
5	76	0.09	10.00	9.10	180	nd	542	175	+	−	Stable	Predominant central
6	71	0.52	3.37	6.25	261	nd	920	112	−	−	Instable	Predominant central
7	78	0.15	10.60	10.20	134	nd	455	157	+	−	Stable	Predominant central

LogMAR best-corrected visual acuity; mean central and total retinal sensitivity (mCRS and mTRS) in dB; LMH diameters and central foveal thickness (CFT) in microns; interdigitation zone and ellipsoid zone (IZ/EZ) and the external limiting membrane (ELM).

**Table 3:** Statistical analysis of demographical characteristics and functional and morphological parameters, considered for all patients and according to tractional and degenerative LMH groups. LogMAR best-corrected visual acuity; mean central and total retinal sensitivity (mCRS and mTRS) in dB; LMH diameters and central foveal thickness (CFT) in microns.

	All patients	Morphology		*p* value
		Tractional LMH	Degenerative LMH	
N	18	11	7	—
Age	72.5 (68.0; 76.0)	73.0 (68.0; 76.0)	71.0 (70.0; 78.0)	0.61^a^
BCVA	0.09 (0.0; 0.15)	0.0 (0.0; 0.09)	0.15 (0.09; 0.52)	**0.03** ^**a**^
mCRS	13.5 (10.3; 16.5)	16.2 (14.2; 17.7)	10.0 (7.5; 11.8)	**0.0008** ^**a**^
mTRS	15.2 (12.5; 16.3)	15.9 (15.5; 17.0)	10.2 (8.5; 13.8)	**0.0001** ^**a**^
LMH depth	235.5 (190.0; 263.0)	257.0 (205.0; 278.0)	190.0 (169.0; 249.0)	0.06^a^
LMH diameter	539.5 (397.0; 650; 0)	429.0 (314.0; 620.0)	653.0 (455.0; 750.0)	**0.04** ^**a**^
CFT	164.5 (157.0; 185.0)	170.0 (160.0; 186.0)	157.0 (112.0; 175.0)	0.12^a^
Stable fixation, *n* (%)	12 (66.7%)	9 (81.8%)	3 (42.9%)	0.14^b^
Predominantly central fixation status, *n* (%)	15 (83.3%)	10 (90.9%)	5 (71.4%)	0.53^b^

Median (25° percentile; 75° percentile) for continuous variables; *n* (%) for categorical variables. ^a^Wilcoxon rank test. ^b^Fisher's exact test.

**Table 4:** Statistical analysis of demographical characteristics and functional and morphological parameters, considered for all patients and according to IZ/EZ-ELM alteration groups. LogMAR best-corrected visual acuity; mean central and total retinal sensitivity (mCRS and mTRS) in dB; LMH diameters and central foveal thickness (CFT) in microns. Interdigitation zone and ellipsoid zone (IZ/EZ) and the external limiting membrane (ELM).

	All patients	IZ/EZ-ELM integrity		*p* value
		No alteration	Layers alteration	
N	18	10	8	—
Age	72.5 (68.0; 76.0)	72.5 (68.0; 75.0)	73.5 (70.5; 77.0)	0.56^a^
BCVA	0.09 (0.0; 0.15)	0.0 (0.0; 0.09)	0.15 (0.07; 0.41)	**0.02** ^**a**^
mCRS	13.5 (10.3; 16.5)	16.4 (15.7; 17.7)	10.1 (7.8; 11.2)	**0.0001** ^**a**^
mTRS	15.2 (12.5; 16.3)	15.8 (14.9; 16.7)	12.0 (8.8; 14.8)	**0.04** ^**a**^
LMH depth	235.5 (190.0; 263.0)	255.5 (202.0; 278.0)	219.5 (185.0; 255.0)	0.36^a^
LMH diameter	539.5 (397.0; 650; 0)	569.0 (410.0; 634.0)	498.5 (371.5; 714.0)	0.70^a^
CFT	164.5 (157.0; 185.0)	173.0 (162.0; 186.0)	153.0 (125.0; 169.5)	**0.02** ^**a**^
Stable fixation, *n* (%)	12 (66.7%)	8 (80.0%)	4 (50.0%)	0.32^b^
Predominantly central fixation status, *n* (%)	15 (83.3%)	9 (90.0%)	6 (75.0%)	0.56^b^

Median (25° percentile; 75° percentile) for continuous variables; *n* (%) for categorical variables. ^a^Wilcoxon rank test. ^b^Fisher's exact test.

## Data Availability

The statistical data used to support the findings of this study are available from the corresponding author upon request.
